# Intelligent Perception System of Robot Visual Servo for Complex Industrial Environment

**DOI:** 10.3390/s20247121

**Published:** 2020-12-11

**Authors:** Yongchao Luo, Shipeng Li, Di Li

**Affiliations:** 1 School of Electrical Engineering, Guangzhou College, South China University of Technology, Guangzhou 510006, China; luoyc@gcu.edu.cn; 2 School of Mechanical and Automotive Engineering, South China University of Technology, Guangzhou 510641, China; itdili@scut.edu.cn

**Keywords:** deep learning, visual servoing, industry robot, intelligence perception

## Abstract

Robot control based on visual information perception is a hot topic in the industrial robot domain and makes robots capable of doing more things in a complex environment. However, complex visual background in an industrial environment brings great difficulties in recognizing the target image, especially when a target is small or far from the sensor. Therefore, target recognition is the first problem that should be addressed in a visual servo system. This paper considers common complex constraints in industrial environments and proposes a You Only Look Once Version 2 Region of Interest (YOLO-v2-ROI) neural network image processing algorithm based on machine learning. The proposed algorithm combines the advantages of YOLO (You Only Look Once) rapid detection with effective identification of ROI (Region of Interest) pooling structure, which can quickly locate and identify different objects in different fields of view. This method can also lead the robot vision system to recognize and classify a target object automatically, improve robot vision system efficiency, avoid blind movement, and reduce the calculation load. The proposed algorithm is verified by experiments. The experimental result shows that the learning algorithm constructed in this paper has real-time image-detection speed and demonstrates strong adaptability and recognition ability when processing images with complex backgrounds, such as different backgrounds, lighting, or perspectives. In addition, this algorithm can also effectively identify and locate visual targets, which improves the environmental adaptability of a visual servo system

## 1. Introduction

Robot visual servo is a type of technology that uses visual sensors to obtain environmental information and then give the corresponding movement command to the robot controller, so it can be considered as an imitation of human eyes and arms [1]. As the traditional robot control technology has been maturing, using vision or touch to perceive the environment to enable a robot to obtain more action instructions has become a hotspot in robot control research [2,3,4]. The vision-based environment perception technology mainly focuses on two problems: target detection and target segmentation.

In the visual servo process, mapping between point feature image coordinates and robot velocity can be obtained directly through the analysis of the changing velocity and robot kinematics; thus, point features become the preferred choice for image feature extraction in most vision servo systems. However, in the process of target recognition, because of environmental constraints, in many cases, traditional image algorithms cannot complete the recognition and extraction of a target image, which ultimately leads to the failure of the servo task, so the first task of visual servo is effective recognition of the target object. Traditional robot vision target detection algorithms generally perform four tasks—image preprocessing, candidate area selection, feature extraction, and final classification—to complete a task. Image preprocessing includes many processes, such as denoising, cropping, and scaling. Candidate area selection is to adopt a certain area selection algorithm to select areas of different sizes, lengths, and width ratios as the target area for detection. Feature selection is to use specific algorithms to extract feature vectors from each candidate area, such as HARR, SIFT, and SURF [5,6]. The last task is to send the feature vector obtained by feature extraction to a pretrained classifier, such as a support vector machine (SVM) and Adaboost [7], for classification, to determine whether the current area contains the target of interest and determine its category. Traditional target detection algorithms rely on artificially designed features to extract image information, but due to the diversity of objects to be detected in the industrial environment, image illumination changes, and occlusion problems, the generalization ability of artificially designed features is poor, so the traditional target detection algorithms difficultly meet the needs of various detection tasks.

With the emergence and wide application of deep learning, detection algorithms have rapidly improved their performance using deep-learning tools, and detection algorithms based on deep learning have become the mainstream of detection algorithms. The Overfeat algorithm proposed by Sermanet et al. [8] uses convolutional networks to extract image features, performs target detection on each sliding window, and finally, completes the precise positioning task of the target. Girshick et al. [9] used a selective search algorithm M to extract candidate frames [10]; then, extracted features using the convolutional network finally employed the SVM to classify feature vectors. Although region-convolutional neural networks (R-CNN) significantly surpass traditional algorithms in detection performance, there are problems of computational redundancy and complex steps. In order to solve these problems, He et al. [11] proposed a SPP (spatial pyramid pooling) structure that does not require feature extraction, which greatly improves the computational efficiency, but its SPP-Net requires multistep training and multiple SVM classifiers, causing the problems of large memory and time consumption. Girshick et al. [9] used the ROI (region of interest) pooling structure to replace the spatial pyramid structure, Softmax to replace the SVM, and a multitask loss function to train the classification and boundary regression of the detection frame uniformly, achieving great improvements in both accuracy and speed [12]. The Mask R-CNN algorithm proposed by He et al. [11], etc. employs a target mask as the output on the basis of the fast R-CNN [13], further improving the detection accuracy, but the detection speed of this algorithm is slow. In order to meet the requirements for real-time detection performance, You Only Look Once Version 2 (YOLO-v2) and the Single-Shot MultiBox Detector (SSD) based on regression have been proposed [14], and compared to the detection algorithm based on candidate frames, the speed has been greatly improved, but the detection accuracy has been poor for larger or smaller targets [15,16].

In terms of target segmentation, traditional image segmentation refers to the separation of specific foreground objects and the background, and traditional image segmentation methods include the segmentation methods based on the threshold, region, edge, and graph theory. However, there are problems, such as unstable segmentation, relying on a manual design of shallow features, being prone to a large number of broken edges, and poor segmentation effects. The emergence of deep-learning image segmentation algorithms has solved the problems of traditional segmentation algorithms. For instance, Long et al. [17] proposed a full convolutional neural network (FCNN) [17], where the fully connected layer in the common image classification network was replaced with a convolutional layer, and deconvolution was used to generate a segmented image of the same size as the original picture directly, realizing end-to-end image semantic segmentation. The SegNet convolutional network adopts a one-to-one corresponding encoder–decoder structure, where the encoder performs the maximum pooling operation and records the index position of the pooling, which is then used by the decoder to perform nonlinear sampling; by using this structure [18], the SegNet effectively improves segmentation accuracy [19]. The U-Net network can be used for the binary semantic segmentation of medical images, and its main advantage is a fewer number of model parameters, which makes it able to complete training on a small-scale dataset, achieving good results [20]. In addition, the DeepLab series of networks replace ordinary convolution operations, and the addition of conditional random fields (CRF) by using hole convolution improves the segmentation accuracy [21].

The image segmentation algorithm is an important component of a visual perception system. In recent years, significant progress in image segmentation has been made with the help of deep-learning tools, but how to improve segmentation fineness, reduce the number of parameters and calculations, and increase the inference speed to achieve specific applications still needs to be further explored [22]. Inspired by previous research on image recognition and measurement results of the above-mentioned deep-learning algorithms, this paper considers common constraints in industrial environments and proposes an optimized YOLO-v2-ROI neural network algorithm. The YOLO prediction process is simple and fast, and some versions can reach 150 frames/s, so YOLO can realize real-time detections. Unlike the sliding window method and the region proposal-based method, YOLO can use the full image information in the training and prediction processes, and the fast R-CNN detection method can falsely detect the plaques in the background as a target, because fast R-CNN cannot see the global image during detection [23]. Compared to the Fast R-CNN, YOLO has a half-lower background prediction error rate, and YOLO can learn the generalizable representation of a target, which has certain universality. The YOLO is trained using natural pictures, and then, the artistic images are used in the prediction process. Besides, YOLO is much more accurate than other target detection methods, such as direct part marking (DPM) and R-CNN.

**Figure 1:**
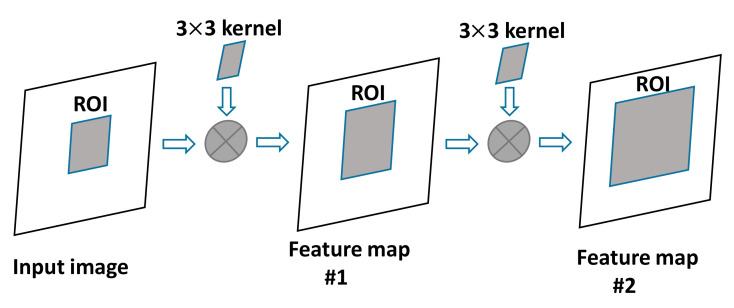
Region of Interest (ROI) changes without sampling.

However, in terms of accuracy, the YOLO algorithm still lags behind the most advanced detection systems [24]. Although it can quickly identify objects in an image, it is difficult to locate certain objects accurately, especially small objects. The ROI pooling is a pooling operation for ROI that has been widely used in the research field of object detection [25]. The purpose of this operation is to use the pooling method to obtain a fixed-size output feature map for ROI of different sizes in the input feature map. The function of ROI pooling is to resize different feature maps into a uniform size, and in this way, the pretrained fully connected layer parameters can be used to improve the detection efficiency of the target extraction algorithm, as shown in Figure 1.

Figure 1 shows an example of the ROI size change of a continuous convolutional layer without sampling. In Figure 1, the left side represents the input of the YOLO network; ROI is displayed as a shadow area, a 3 × 3 kernel is used for convolution, and the shading in the figure represents the new ROI generated by the feature map. This paper combines common features of the two algorithms to locate and identify different objects in the field of view quickly. The control robot vision system automatically recognizes and classifies the target object, improving the work efficiency, avoiding blind movement, and reducing the computational load.

The main contributions of this article are as follows:

1. The visual constraint conditions in the actual production environment are evaluated, and various constraint conditions are quickly identified based on visual characteristics, which effectively improves the detection accuracy while ensuring the real-time performance of the calculations.
2. By using YOLO-v2 as the main network model in combination with the ROI pooling structure, densely connected convolutional networks, and embedded deep-dense modules, the proposed network can make full use of high-resolution features of an image to realize the multiplexing and fusion of the shallow and deep features of the image.
3. Based on the requirements for the real-time processing of environmental information by environmental perception systems, this paper designs a joint architecture for detection and segmentation; i.e., target detection and semantic segmentation share the same feature extraction network through joint training while reducing the inference time, thus effectively improving the performance of subtasks.

The rest of the paper is organized as follows. Section 2 classifies and evaluates constraints in the industrial environment. Section 3 introduces the proposed image target detection model. Section 4 presents the experimental results. Section 5 reviews and summarizes the paper.

## 2. Classification of Visual Objects in the Industrial Environment

There are many types of images in the field of vision of visual systems in the industrial environment, especially for a mobile robot arm installed on an automated guided vehicle (AGV) [22]; namely, color, shape, size, and texture of a target object are similar to a certain extent, so how to reasonably classify targets is a prerequisite for realizing the function of robot intelligent perception in a visual servo system. In order to facilitate the realization of intelligent perception system functions, this paper studied target shape, color, size, unevenness, texture, and other factors and selected objects with similar features that were likely to cause interference to target object detection. According to the difference between the object and target object in different dimensions within the camera’s field of view, to distinguish interfering objects from target objects, the robot arm performs the next action according to the judgment of the intelligent perception system. Characteristics of the target and interference objects are given in Table 1, the setting of the interference target includes the interference factors that can be encountered in the process of target recognition under normal circumstances, including only different textures, only different colors, only different edges, only different shapes, etc. These factors will cause great difficulty to target identification.

**Figure 2:**
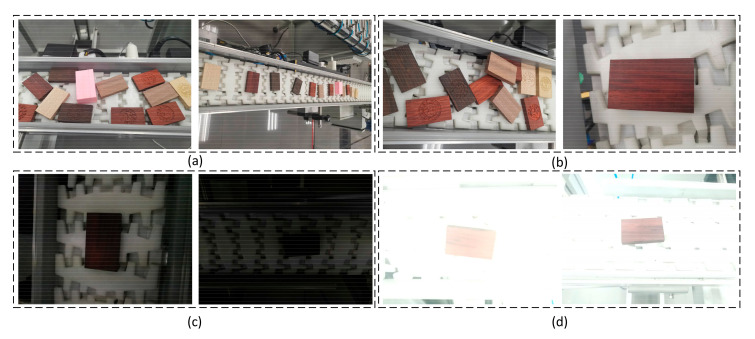
Target training images under different environmental conditions. The picture contains (**a**) different interference backgrounds, (**b**) different visual field distances, (**c**,**d**) are different light intensities.

In order to detect a target effectively and quickly under different environmental conditions, the experiment process takes into account possible effects of different angles, viewing angles, distances, illumination, occlusion, and other related factors of the target object. The target images used for training under different environmental conditions are shown in Figure 2.

In order to increase the sample size of the network training and expand the existing samples, the image data enhancement tool Image-Data-Generator provided by Keras [26] was used in the experiments to perform horizontal mirror flip, random rotation, cropping, scaling, and other processing on the training samples. Finally, 2076 pictures were obtained and used as the training set. The LabelImag tool [27] was used to mark the rectangular coordinate area of a real target object in all pictures, and the obtained coordinate information was saved in the corresponding image.

It should be noted that the algorithm matches according to different characteristics, and when there is a feature point that cannot be matched, it is judged as an interference object, and matching of other features is omitted to reduce the computational load.

### Basic Interaction Matrix of Visual Servo

In the actual robotic vision control process, especially in industrial environment applications, camera calibration is required before the task begins. This calibration requires mutual conversion between the internal camera parameters, external parameters, camera coordinate system, image coordinate system, robot coordinate system, world coordinate system, target coordinate system, etc. However, the joint velocity is still calculated based on the theory of small hole transformation imaging. In the following, we briefly introduce the basic mathematical model and theory used in this article.

Under the camera coordinate system, the 3-dimensional (3D) point coordinates $A=\left(X,Y,Z\right)$ are projected on the planar image, and the coordinates of the resulting 2-dimensional (2D) points are expressed as $A^{\prime }=\left(x,y\right)$. According to the principle of small hole imaging, the projection relationship between the two points is shown as follows:

(1) $$\left\{\begin{array}{c} x=X/Z=\left(u-c_{u}\right)/fa\\ y=Y/Z=\left(v-c_{v}\right)/f \end{array}\right.$$

**Figure 3:**
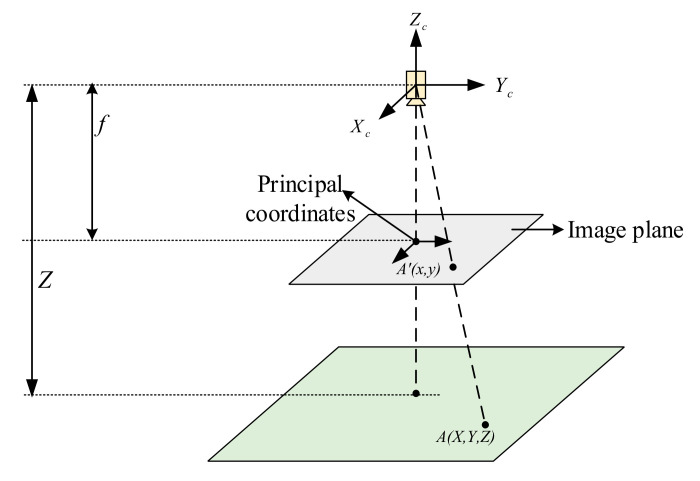
Schematic diagram of the correspondence between the target feature point and camera image plane. Z is the distance from camera to image, f is the camera focal length, $A=\left(X,Y,Z\right)$ is spatial coordinates of image point, $A^{\prime }=\left(x,y\right)$ is the projection of $A=\left(X,Y,Z\right)$ in image plane.

The image point coordinates $m=\left(u,v\right)$ refer to the pixel positions, and $a=\left(c_{u},c_{v},f,a\right)$ is the camera’s internal series of parameters, where $c_{u}$ and $c_{v}$ are the main point coordinates, f is the focal length, and a is the ratio of pixel dimensions. The projection relationship is shown in Figure 3.

The derivative of Equation (1) can be written as follows:

(2) $$\left\{\begin{array}{c} \dot{x}=\dot{X}/Z-X\dot{Z}/Z^{2}=\left(\dot{X}-x\dot{Z}\right)/Z\\ \dot{y}=\dot{Y}/Z-Y\dot{Z}/Z^{2}=\left(\dot{Y}-y\dot{Z}\right)/Z \end{array}\right.$$

It is known that the motion of a point in space can be divided into rotational and translation motions, and its relationship with the velocity of the camera, attached to the end of robot, can be expressed by the following expression:

(3) $$\dot{X}=-v_{c}-\omega_{c}\times X\Leftrightarrow \left\{\begin{array}{c} \dot{X}=-v_{x}-\omega_{y}Z+\omega_{z}Y\\ \dot{Y}=-v_{y}-\omega_{z}X+\omega_{x}Z\\ \dot{Z}=-v_{z}-\omega_{x}Y+\omega_{y}X \end{array}\right.$$

Based on Equations (2) and (3), we can obtain

(4) $$\left\{\begin{array}{c} \dot{x}=-v_{x}/Z+xv_{z}/Z+xy\omega_{x}-\left(1+x^{2}\right)\omega_{y}+y\omega_{z}\\ \dot{y}=-v_{y}/Z+yv_{z}/Z+\left(1+y^{2}\right)\omega_{x}-xy\omega_{y}-x\omega_{z} \end{array}\right.$$

Furthermore, the above equation set can be written as

(5) $$\dot{Χ}=L_{x}V_{c}$$

From Equations (4) and (5), the interaction matrix $L_{x}$ can be expressed as follows:

(6) $$L_{x}=\left[\begin{array}{cccccc} \frac{-1}{Z} & 0 & \frac{x}{Z} & xy & -\left(1+x^{2}\right) & y\\ 0 & \frac{-1}{Z} & \frac{y}{Z} & 1+y^{2} & -xy & -x \end{array}\right]$$

In Equation (6), Z is the depth between the 2D plane point and camera coordinates. It can be seen that, for an ordinary 6 degrees of freedom (DOF) robot, only three interaction points $x=\left(x_{1},x_{2}{,x}_{3}\right)$ should be theoretically stacked to calculate the joint velocity of the robot. The expression of the Jacobian interaction matrix that connects the image features and joint velocity of the robot is as follows:

(7) $$Lx=\left[\begin{array}{c} L_{x_{1}}\\ \vdots \\ L_{x_{n}} \end{array}\right]$$

Considering $V_{c}\;$ as the input to the robot controller and if we would like, for instance, to try to ensure an exponential decoupled decrease of the error, we obtain:

(8) $$V_{c}=-\lambda L_{x}^{+}e$$

Equation (7) is the representation of the composite Jacobian matrix J described below. Therefore, in the experimental section, according to Equations (6)–(8), we can calculate the joint speed of the robotic arm based on four points. As shown in experiment part, once the target block in the frame is recognized, the joint velocity of the robot can be calculated according to corner points of the four corners on the front of the block (not in the same straight line).

## 3. Object Detection Model

YOLO-v2 is a detection algorithm based on direct regression, which is not required to select candidate frames explicitly [28]. The convolutional network is used to determine the category and location of the target of interest directly. Additionally, the training and inference processes are much faster than those of the detection algorithm based on candidate boxes and can meet the requirements for timeliness. However, there is still a certain gap in the accuracy between YOLO-v2 and the detection method based on the candidate frame [29], and this gap is usually considered to be caused by the category prediction and position regression in the subsequent convolutional layer and loss of high-resolution information. The target detection algorithm based on the candidate frames has more advantages in accuracy than the target detection algorithm based on direct regression, but its speed is slower. The main reason for the difference between the two algorithms is that the detection algorithm based on the candidate frame is before the final category classification and position regression. Besides, preclassification of the foreground and background is performed when extracting candidate frames, which provides good prior knowledge for the final classification and regression, but the additional storage and computational overhead of the regional candidate network make the detection speed incomparable with that of the detection algorithms based on direct regression. Therefore, this paper proposes a direct regression idea fused with the YOLO-v2 and the target detection algorithm based on the ROI extraction of detailed features, which implements a cross-layer ROI pooling structure in the process of direct regression and uses high-resolution features to achieve better detection results.

### 3.1. YOLO v2 Dense Detection Model

**Figure 4:**
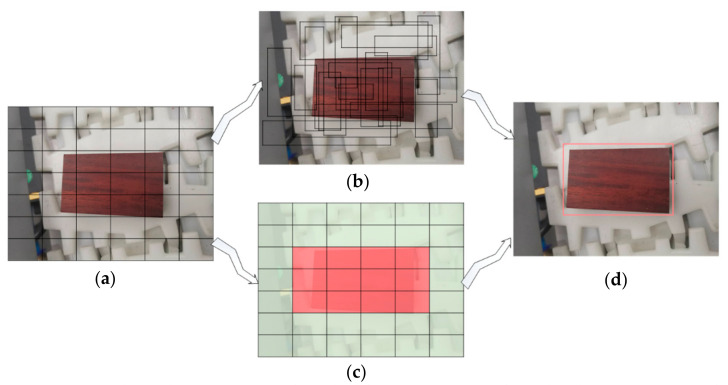
You Only Look Once (YOLO) algorithm detection process adopted in this paper. (**a**) Divided the input image into *C × C* network. (**b**) Target-predicting candidate boxes and confidence. (**c**) Target category probability map. (**d**) Complete target detection.

The network structure of the YOLO-v2 model is shown in Figure 4; these are the four key and necessary steps for algorithm implementation.

First, YOLO-v2 divides the input image into C×C grids, and if the coordinates of the center of an object fall into a grid, then the grid is responsible for detecting this object. The information of the sliding window is represented by a set consisting of five parameters, $T\left(x,y,w,h,confidence\right)$, where x and y denote the abscissa and ordinate of the confidence center position of the detection object predicted by the current grid, respectively; w and h denote the width and height of the sliding window, respectively; confidence reflects whether the current sliding window contains the estimated probability of the detection object and its prediction accuracy, which is expressed as:

(9) $$confidence=P\left(object\right)\times IOU_{pred}^{truth}=\left\{\begin{array}{c} IOU_{pred}^{truth}\;\;\;have\;target,\\ 0\;\;\;\;\;\;\;\;\;\;\;\;\;\;\;\;no\;target, \end{array}\right.$$

where $P\left(object\right)$ represents the probability that the sliding window contains the detection object, and $IOU_{pred}^{truth}$ indicates the overlap area between the sliding window and the real detection object area; if there is a target in the cell, then $P\left(object\right)$ is equal to one, and the confidence is $IOU_{pred}^{truth}$; otherwise, the $P\left(object\right)$ and confidence are both equal to zero. In the test, the confidence score of a specific category in the candidate box is calculated by:

(10) $$P\left(class_{i}|object\right)\times P\left(object\right)\times IOU_{pred}^{truth}=P\left(class_{i}\right)\times IOU_{pred}^{truth}.$$

The value of Equation (10) represents the probability that a particular category appears in a candidate box and the probability that the candidate box matches the target object. In order to improve the detection accuracy, YOLO-v2 adopts a series of improvement measures, including the normalization processing, high-resolution classifier, introduction of the anchor mechanism, dimensional clustering, direct position prediction, fine-grained features, multiscale training, and other techniques. In order to increase the speed, YOLO-v2 uses a relatively simple Darknet-19 network. In order to improve the classification performance further, joint training methods can also be used, combined with the word tree and other methods, to continue expanding the detection types of YOLO-v2.

**Figure 5:**
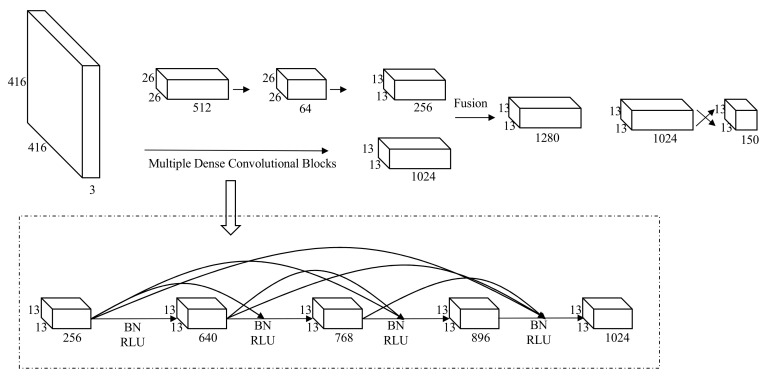
Scene segmentation graphs obtained by different algorithms. RLU: rectified linear units and BN: normalization.

After the image features are extracted by 20 convolutional layers and five pooling layers in the YOLO-v2 network structure, the deep layer hardly uses shallow information, the utilization of high-resolution shallow features is significantly reduced, and features on the corresponding feature map are often difficult to fully train, thereby affecting the detection accuracy. In order to make full use of the high-resolution features, the multiplexing and fusion of features are realized by embedding the deep-dense modules. This paper introduces a densely connected convolutional network to improve the YOLO-v2 network structure, named the YOLO v2-DENSE network, which is shown in Figure 5.

The specific steps of this paper are as follows:

1. In the YOLO v2-DENSE network, use the 21-layer feature map $x_{0}$ as the input of $H_{1};$ after normalization and function active rectified linear units (RLU), convolve into 256 feature maps with 256 1 × 1 convolution kernels; then, through normalization and RLU operation, use 128 3 × 3 convolution kernels to obtain 128 feature maps $x_{1}$; finally, stitch $x_{0}$ and $x_{1}$ into 640 feature maps, and use $\left[x_{0},\;x_{1}\right]$ as the input of $H_{2}$.
2. $H_{2}$ after normalization and activation function RLU convolve into 256 feature maps with 256 1 × 1 convolution kernels; then, through normalization and RLU operation, use 128 3 × 3 convolution kernels to obtain 128 feature maps $x_{2}$; then, merge $x_{0},x_{1},$ and $x_{2}$ into 768 feature maps, and use $\left[x_{0},x_{1},x_{2}\right]$ as the input of $H_{3}$.
3. By analogy, the deep-feature map of 13×13×1024  channels are obtained. The DENSE Net makes the input of layer l directly affect all subsequent layers, and its output is expressed as:
  (11) $$x_{l}=x_{l}\left(\left[x_{l},\;x_{l},\;\cdots ,x_{l-1}\right]\right),\;l=1,2,\cdots$$
  where $x_{0}$ denotes the input feature map of the module, $x_{1}$ represents the output of the first layer, $\left(\left[x_{0},\;x_{1},\cdots ,x_{l-1}\right]\right)$ represents the connection of $x_{0},\;x_{1},\cdots ,x_{l-1}$; $H_{l}\left(\cdot \right)$ denotes the combined operation of normalization (BN), activation function RLU, and convolution and realizes the first layer nonlinear transformation. The operation $H_{l}\left(\cdot \right)$ used in this article is as follows: $BN\to RLU\to Conv\left(1\times 1\right)\to BN\to RLU\to Conv\left(1\times 1\right)$, where $Conv\left(n\times n\right)$ indicates that the size of the convolution kernel of the convolution operation is n×n. Since each layer contains the output information of all previous layers, the problem of gradient disappearance caused by the increase in the depth of the deep convolutional neural network is solved to a large extent, improving the target detection effect.

### 3.2. YOLO-v2 Algorithm Architecture Integrating ROI

As previously mentioned, there is a certain gap in the extraction accuracy of a target image between the detection algorithm based on direct regression and that based on the candidate frames. Therefore, this paper adopts a target detection algorithm that combines the ideas of YOLO direct regression and detailed feature extraction based on candidate frames and uses higher resolution to achieve better detection results. As shown in Figure 1, after convolution, the ROI in the feature map is larger than ROI in the input image, which is because the convolution operation will cause a single pixel in the input image to affect the value of nine pixels (3 × 3 pixels) in the output image. In Figure 1, the ROI of feature map 2 is greater than the ROI of the previous layer, and since the convolution operation continues to the end of the network, the ROI will become larger and larger and will eventually be the same as the input image; this means that all data of the input image will be treated as an ROI, which will result in a reduction in the calculation accuracy of the final ROI. Therefore, after the convolution operation, it is necessary to derive a suitable ROI selection plan.

**Figure 6:**
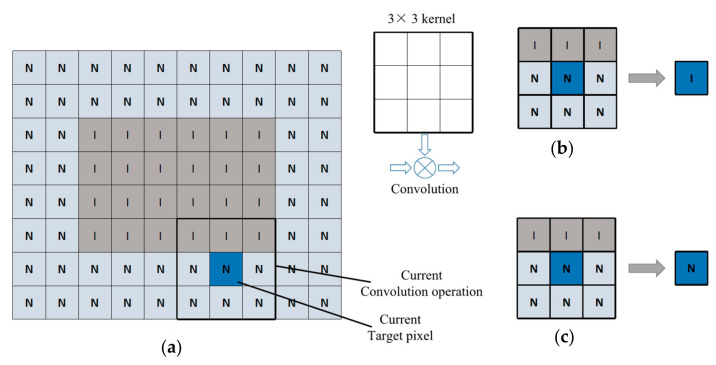
(**a**) The example of the ROI and convolution operations. (**b**) The ROI output of TH0. (**c**) The output ROI of TH3.

An example of ROI determined by a convolutional layer is presented in Figure 6, where each box corresponds to a pixel, “N” and “I” represent RONI (region of not interest) and ROI data, respectively, and the kernel size is 3 × 3.

If the amount of ROI data in the kernel window is greater than the predetermined threshold, the output should be ROI. In Figure 6a, the thick square in the bottom-right corner of the input image indicates the sliding window operation of the current convolution. The output of the convolution operation is regarded as ROI data, and the output after convolution depends on the amount of ROI data in the input window. In Figure 6, three pieces of data are in ROI, and the remaining six are in RONI. The predefined threshold is used to determine that the ROI area of the output layer is in the output layer. When the data value is 0, 1, or 2, it is output as the ROI area, and the data values over 2 will be output as the RONI area [23]. In Figure 6b, when the threshold is zero, it means that the kernel window contains at least one ROI data, and the output is ROI. Figure 6b shows that, when the threshold condition is zero (TH0), the data adjacent to the boundary between RONI and ROI are transformed into ROI data, so the ROI size obtained by the convolutional layer is larger than the input ROI size. Figure 6c shows that, when the threshold condition is three (TH3), the ROI will not increase, because once the ROI area is output, the operation of generating data in the RONI area is avoided; additionally, the amount of calculation is reduced, and it will not affect the calculation load of the system. Each layer processes only the operations generated by the ROI data and passes the results to the next layer.

**Figure 7:**
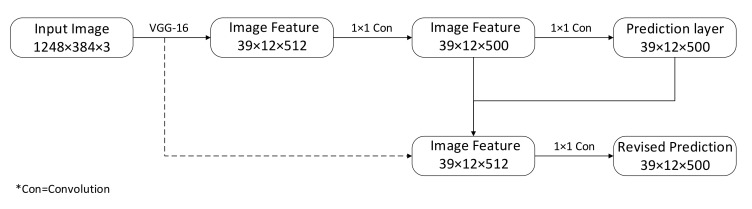
The proposed structure. VGG: visual geometry group.

The target detection algorithm used in this study consists of two parts: encoder and decoder. The encoder extracts image features, and the decoder determines the category and coordinates according to the features extracted by the encoder. Based on the comparison of common convolution models, the visual geometry group (VGG) network is a feature extractor with excellent performance, and the top-5 error rate of the ImageNet image classification tasks is lower. Although the accuracy of the convolutional models, such as GoogleNet and ResNet, on this dataset is still better than that of the VGG model, in multiple transfer learning tasks, the VGG model performs better. Therefore, this paper uses the first 11 layers of the VGG-16 network model as the encoder to extract image information—that is, the input image—to obtain the output of the 5th layer of the VGG-16 network pool and send it to decoder. The decoder is similar to YOLO-v2; first, six channels of 36 × 12 are directly generated through two-layer 1×1 convolution, of which the first two channels predict whether there are objects of interest in the grid, and the last four channels predict the coordinates of the candidate frames in the grid area. However, since part of the image detail information is inevitably lost in the feature extraction process, the prediction results produced in this way are not highly accurate, and in order to improve the positioning accuracy of the detection frame, the ROI pooling in the detection algorithm based on the candidate frame is used to fuse high-resolution features. Similar to the region proposal network RPN network, the detection frame obtained by a rough estimation is first mapped to the high-resolution VGG feature map, and then, ROI pooling is used to convert the mapped feature map into a tensor of the same size as a rough prediction; finally, concatenate the tensors and use 1×1 convolution to produce more accurate predictions, as shown in Figure 7.

## 4. Experimental Results Analysis

### 4.1. Experimental Platform Construction

**Figure 8:**
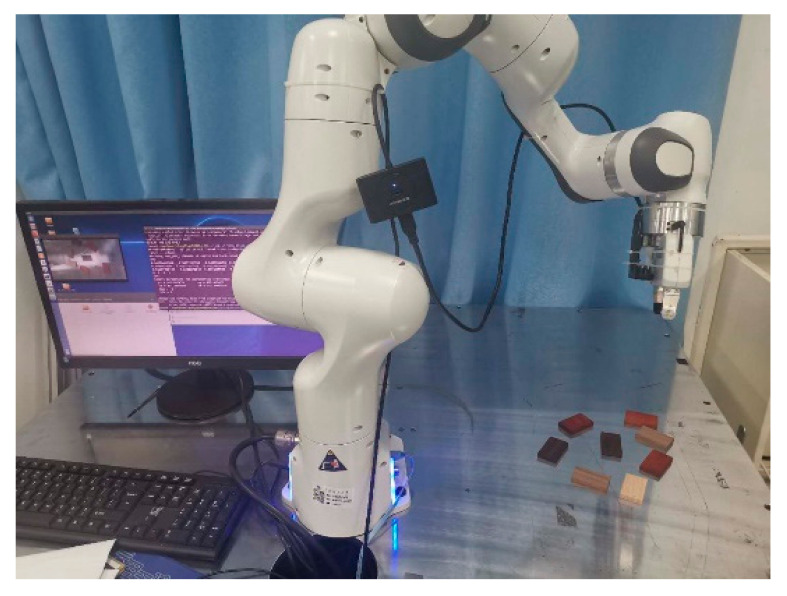
Experimental platform.

In order to test the effectiveness of the proposed algorithm, an industrial robot visual servo platform was built for verification in the laboratory. As shown in Figure 8, the experimental platform included the seven-axis collaborative robot Franka Panda (Franka Emika GmbH, Munich, Germany), depth camera Intel Realsense SR300 (Intel Corporation, Santa Clara, CA, USA), and operate system is Linux 16.04 (Canonical Group Ltd., London, UK). Other components included the OpenCV 4.1(Palo Alto, CA, USA), TensorFlow 2.0 [30], LabelImg [27] Caffe [31], and many others.

**Figure 9:**
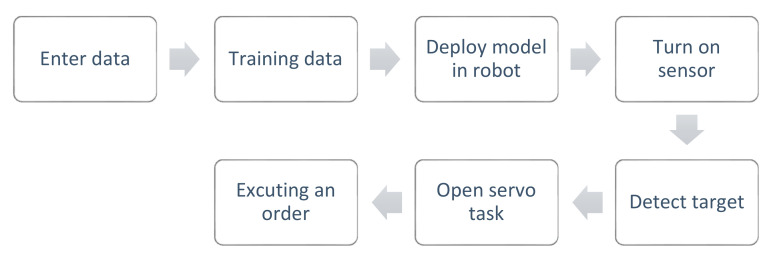
The workflow of the test system.

The overall workflow of the test system is displayed in Figure 8, and it was as follows. First, the dataset was entered, and the YOLO-v2-ROI network model was trained for target detection and classification on the server. Then, the trained model was deployed to the robot ROI, and the vision sensor was turned on; when the camera detected the presence of a certain target object, the robot was guided to the target position, and the corresponding task was performed according to the system action mode, as shown in Figure 9.

### 4.2. Training Parameters Configuration

Part of the training parameters is shown in Table 2.

Learning is essentially an optimization process that progressively tends to the optimal solution, but how much error is utilized for each update parameter requires a parameter to control, and this parameter is the learning rate in the table above; the learning decay strategy is represented by the step size, and the entire learning process is performed with 5000 sample updates. When training the YOLO-v2-ROI, the initial weights of the network need to be initialized according to some distribution, e.g., Gaussian distribution. The initial weighting operation has a great impact on the final YOLO-v2-ROI performance. The appropriate initial weighting of YOLO-v2-ROI can make the loss function converge faster in the training process to obtain better optimization results, but there are some uncertainties when initializing YOLO-v2-ROI randomly according to a certain type of distribution. The inappropriate initial weights may make the YOLO-v2-ROI loss function fall into a local minimum during the training process and not reach the global optimal state, so we need to solve this problem through the momentum; when the momentum is larger, the energy converted into potential energy is also larger; it is more likely to get rid of the local concave domain into the global concave domain; the purpose of regular weight attenuation is to let the weights decay to a smaller value. The learning rate is a hyperparameter that adjusts the weight of the YOLO-v2-ROI during learning and the loss gradient.

The several training parameters listed in Table 2 generally affected the learning efficiency and convergence rate. Changes in these parameters cannot be seen in recognition results of one single target image, for example, when the learning rate is set too small, the convergence process will become very slow, and when set too high, the gradient may oscillate back and forth around the minimum value, may even fail to converge. For instance, if the learning rate is set to 0.1 and other parameters remain unchanged, the target cannot be detected in the same scenario. Similarly, the role of the momentum is mainly to make the function reach the global optimum state and improve the convergence speed of the attenuation function in the training process. The most important influences of these parameters on the detection results are shown in Table 3: accuracy and detection speed.

### 4.3. Experimental Results and Analysis

**Figure 10:**
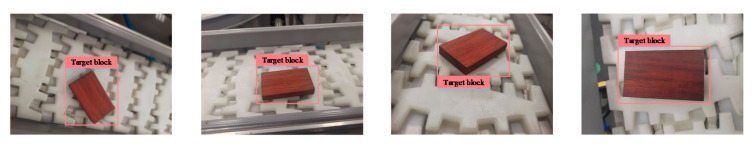
Detection result of the single-target detection.

**Figure 11:**
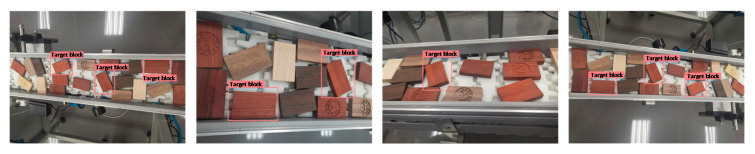
Detection result of the multitarget detection.

The partial detection results of the proposed improved network YOLO-v2-ROI on the test images are presented in Figure 10 and Figure 11.

Figure 10 shows the results of the single-object detection in a complex industrial environment, and Figure 11 shows the results of the multi-object detection in a complex industrial production line environment.

**Figure 12:**
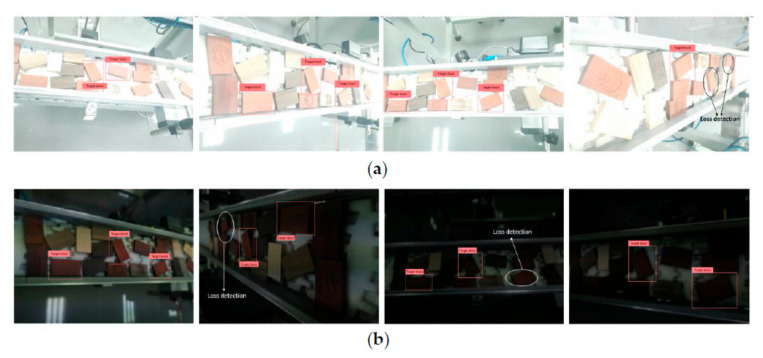
Target recognition results under different ambient light conditions. (**a**) The recognition of the complex background targets under strong illumination. (**b**) The recognition of complex background targets under low illumination.

The test results of the two sets of pictures show that YOLO-v2-ROI can successfully detect the target object, which demonstrates the effectiveness of the detection algorithm. The recognition results of the images under different lighting and shadow conditions are presented in Figure 12a, where it can be seen that, in most cases, the detection accuracy is high, but there is still a certain recognition error. Some of the wrongly recognized pictures are presented in Figure 12b, where it can be seen that there are misjudgments, including misjudgments in color and texture, which caused a wrong detection.

The analysis of the experimental results shows that the number of sample sets is insufficient, the adaptability of the model needs to be improved, and there is a problem of missed detection and misjudgment. Particularly, when colors, shapes, and volumes are similar, it is very difficult to distinguish and detect objects. Thus, it is necessary to distinguish them from the texture or grayscale image further, which may easily lead to missed detection and misjudgment.

**Figure 13:**
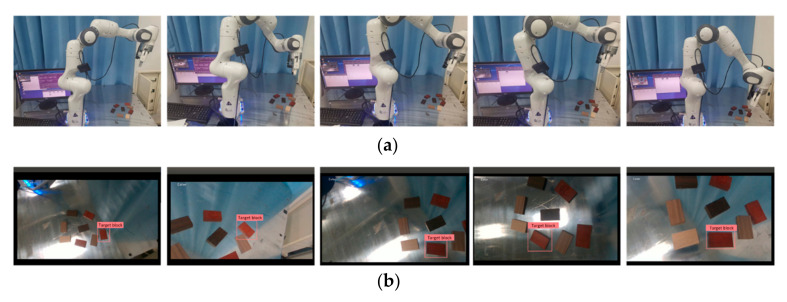
Servo task process of the experiment. (**a**) Relative position of the robot and target at different times. (**b**) Target recognition situation at the corresponding time.

Figure 13 shows the real-time detection process of a target object by the visual servo system. When the servo task started, first, the end speed of the robotic arm was calculated based on the image difference between the current target point and the final target point.

**Figure 14:**
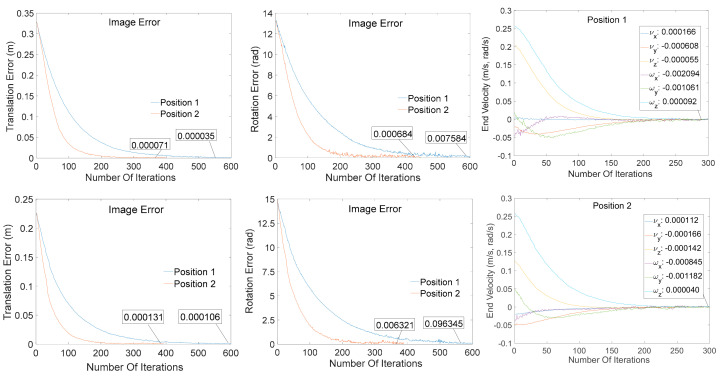
The index data of the robot arms at different positions during the servo process.

Due to the strong environmental stability of the visual detection algorithm, the sensor could detect the target position effectively, even in the complex environment. The image error and the robot arm data of a certain servo task (the end speed consisted of linear and angular velocities) are presented in Figure 14.

According to the presented change in the image error and the speed of the end of the robotic arm, the target image could be effectively detected during the entire vision task, and the process was stable. This result proves the effectiveness of the proposed image algorithm.

In order to illustrate the effectiveness of the proposed algorithm further, for the same object, the image size of the self-built dataset was normalized to 416×416 pixels with the same angles and scales. The detection performances of the original YOLO-v2, YOLO-v2-DENSE, and the proposed improved YOLO-v2-ROI were compared. The comparison results are shown in Table 3, where it can be seen that the long-distance object detection performances of YOLO-v2 and YOLO-v2-DENSE were not good, and for 3D objects, different viewing angles had a great impact on the detection performance. However, the YOLO-v2-ROI showed good distance and environmental adaptability, as shown in Table 3. After detecting 1000 sets of target images, the accuracy rates of the YOLO-v2, YOLO-v2-DENSE, and the proposed YOLO-v2-ROI were 71.45%, 83.51%, and 90.23%, respectively. Hence, the proposed network structure performed the best among all the models, and it could achieve real-time detection. The proposed algorithm can retain more shallow images information and improve the ability to extract target features, achieving stronger adaptability and recognition performance when processing pictures with different illuminations, backgrounds, viewing angles, and resolutions.

## 5. Conclusions

In order to improve the ability of industrial robots to recognize targets in industrial environments, especially long-distance small targets, this paper studies the problems of image recognition and the detection of vision-based robots in interference environments. In the original target detection YOLO-v2-DENSE architecture, the ROI pooling structure model is embedded, and an improved YOLO-v2-ROI network structure is developed. The proposed algorithm makes full use of image feature information while adopting data enhancement and multiscale training strategies to improve the detection accuracy and optimize the real-time detection speed. The proposed algorithm is verified experimentally and compared with two other algorithms. The experimental results show that the overall performance of the proposed YOLO-v2-ROI is better, and the real-time detection speed is faster than those of YOLO-v2 and YOLO-v2-DENSE.

Although the proposed algorithm achieves a certain improvement in the detection accuracy of the targets compared to the existing methods, there are still omissions and errors. The supervised learning method YOLO-v2-ROI has high requirements for the quantity, quality, and diversity of training datasets. In our future research, adversarial generation networks or deep network detection methods based on semi-supervised and unsupervised learning will be used. Additionally, the properties of objects will be considered to realize robot operation suggestions, the servo mode judgment, and other decisions and further improve the autonomous decision-making ability of robots in industrial environments.

In addition, another issue that requires paying attention is the computational cost; it is an important aspect to consider in visual servo, especially when applying deep-learning methods for image detection. The YOLO-v2-ROI image detection algorithm mentioned in this paper takes up more computing costs than the traditional image detection method, especially during target training. For example, for a target image with a resolution of 1280 × 960, as the computer load cannot meet the iteration period of more than 50 Hz, a severe delay will lead to the failure of the servo task. It is the reason why the resolutions of images used in this paper were all below 1280 × 960 (mainly between 1024 × 768 and 800 × 600). Although the computational load problem is not discussed in this paper, we designed an adaptive resource allocation model based on fog computing, and we propose to solve the computational consumption problem by deploying several fog nodes and achieve adaptive resource allocation for the target images of different frequencies and sizes in a servo vision system. The simulation experiments were completed, and a hardware platform was built; this is also one problem that needs to be solved.

## Figures and Tables

**Table 1:** Description of the properties of the targets and interfering objects. YOLO-v2-ROI: You Only Look Once Version 2 Region of Interest.

YOLO-v2-ROI	Match Index	Shape	Color	Texture	Edge	Area
Target object	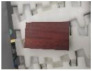	√	√	√	√	√
Interfering object 1	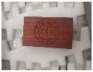	√	√	×	√	√
Interfering object 2	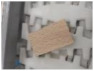	√	×	√	√	√
Interfering object 3	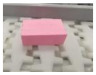	×	×	×	√	√
Interfering object 4	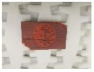	×	√	√	×	×
Interfering object 5	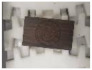	√	×	√	√	√
Interfering object 6	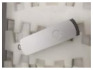	×	×	×	×	×

**Table 2:** Training parameters.

YOLO-v2-ROI	Parameter Value
Learning rate	0.001
Learning attenuation strategy	Steps
Sample update parameters	5000
Momentum	0.76
Weight decay regularization parameter	0.0001
The maximum number of iterations	4500
Learning rate change ratio during the experiment	0.1 0.01 0.001

**Table 3:** Detection effects of different algorithms.

	Enter Size (Pixel)	Iterations Number	Accuracy	Detection Speed (Frame/s)
YOLO-v2	416 × 416	4500	81.45%	25
YOLO-v2-DENSE	416 × 416	4500	83.51%	27
YOLO-v2-ROI	416 × 416	4500	93.23%	36
